# William H Danforth II (1926–2020)

**DOI:** 10.1096/fba.2020-00122

**Published:** 2021-01-04

**Authors:** Philip D. Stahl, Sharon M. Stahl, Ralph A. Bradshaw

**Affiliations:** ^1^ Washington University St. Louis MO USA; ^2^ University of California Irvine CA USA

**FIGURE 1:**
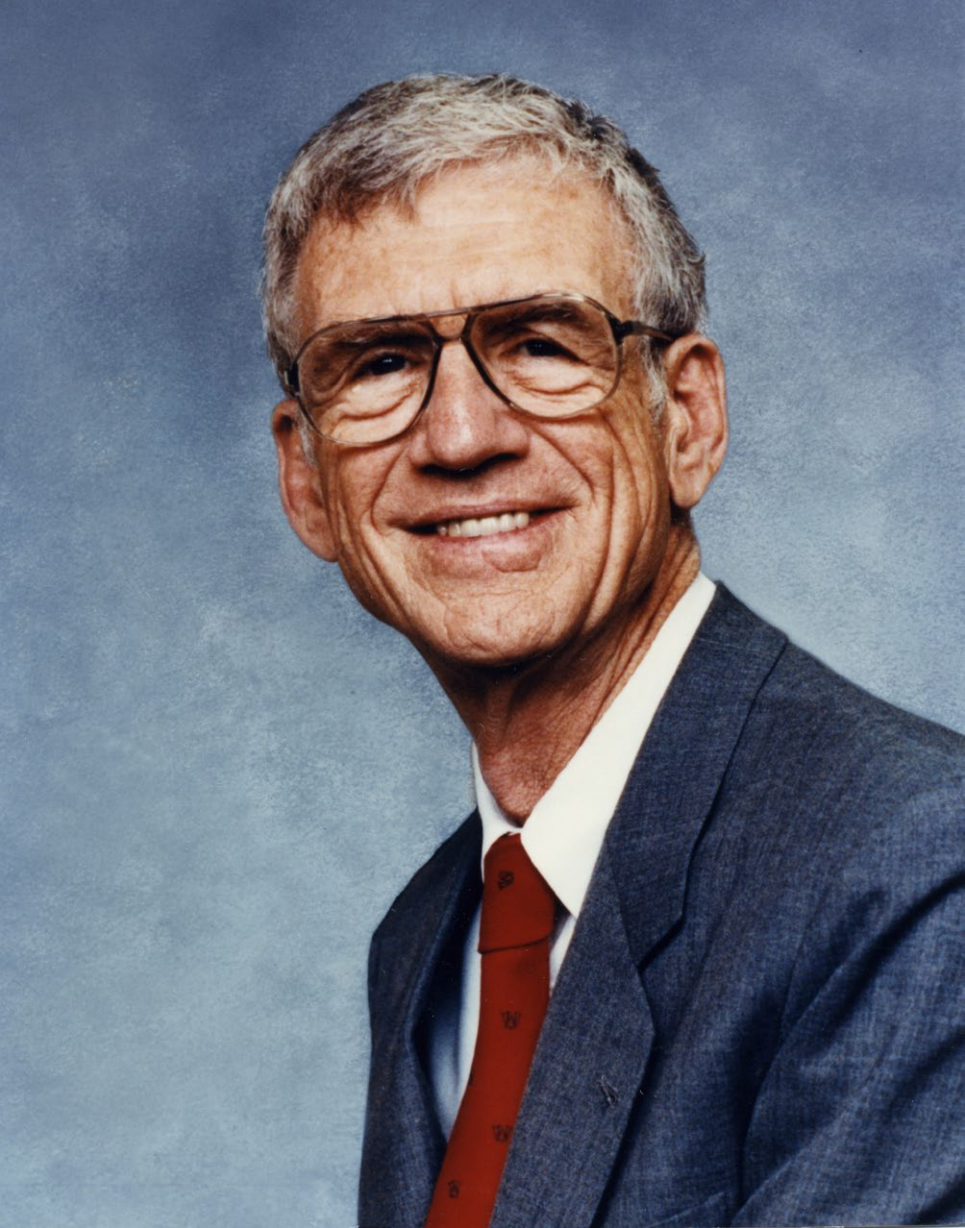
William H. Danforth II. Photo courtesy of Washington University Photographic Services Collection, Washington University Libraries, Department of Special Collections. Used with permission

In the mid‐1960s, Washington University, located in St. Louis, MO, was a small liberal arts school catering mainly to local students that coincidentally had a large, and highly recognized medical school, populated with some of the best physicians and scientists in the world that disproportionally reflected the prestige of the institution. However, beginning in 1965, events would transpire that would set the stage for a 30‐year period of growth and development that brought the two parts closer to parity. The driving force for this transformation was William H. Danforth (Figure 1), a native son and scion of the family that created Ralston Purina, one of the most important economic engines of the area. It began when Danforth, who started his academic career as an instructor at the School of Medicine in the late 1950s, specializing in cardiology, was precipitously elevated to vice‐chancellor for medical affairs and president of the Washington University Medical Center as a compromise candidate to resolve administrative conflicts between the faculty and the overseers of the medical center. This initiated his academic administrative career and his success in this role, due in no small part to his humility, thoughtfulness, attentiveness, empathy, vision, and wisdom that are the foundations for extraordinary and transformative leadership, also presaged his successes with revitalizing the University as a whole when, some six years later, Tom Elliot stepped down as Chancellor of the University and a search committee recommended Danforth to be his successor. Although initial expectations may have been somewhat tempered by his lack of wider administrative experience, his subsequent accomplishments that spanned the next 24 years not only assuaged these concerns but also ultimately defined him as one of the great educators of his generation. Among many other things, he greatly increased the endowment and raised millions of dollars that increased the number of endowed professorships and student scholarships and led to the construction of dozens of buildings, propelling the university from its small local stature to the ranks of the top universities in America.

One of Danforth’s earliest and most notable achievements, but perhaps somewhat lesser known, was his role in the reorganization of biosciences at Washington University. Following on the heels of his promotion, in 1966, the medical school recruited P. Roy Vagelos to take over as head of the Department of Biological Chemistry, where he was to embellish his credentials as an outstanding academic scientist, honed during ten years at the NIH, before eventually joining Merck in 1975 and starting a second, equally illustrious career as a pharmaceutical executive. While at Washington University, Vagelos exerted an enormous impact on revitalizing the School of Medicine, which had, despite a long and distinguished history, begun to lose some momentum. Not the least of his achievements was an ambitious plan he conceived for an entirely new and innovative academic structure, the Division of Biology and Biomedical Sciences (DBBS). Working with Danforth, they put this plan into action, which transformed undergraduate and graduate education in these areas not only at Washington University but also eventually nationwide.

The division structure affected many aspects of teaching and administration at the university and served to help bridge the geographical separation of the two principal campuses. The undergraduate campus of the university (known then as the “Hilltop” campus and renamed in 2006, the Danforth Campus) is situated on the west end of Forest Park, a large city park (~1.5 times larger than Central Park in New York City), while the medical campus is on the east end anchored by Barnes Hospital among other clinical and research facilities. In the early 1970s, the medical school considerably exceeded the undergraduate campus in research facilities, resources, and personnel but had only limited exposure to graduate students and essentially none with undergraduates. Danforth was attracted to the division plan because it was a way of utilizing the full talent of the university to meet its teaching and instructional obligations using existing faculty. The restructuring of the biology and biomedical science programs, which principally involved the biology department on the Hilltop campus and the basic science departments of the medical school included a plan for graduate education that was predicated on providing resources to communities of faculty representing multiple departments and a shared interest in a particular discipline, rather than the traditional approach of recruitment and oversight resting within individual departments. Under this division structure, graduate training programs transcended departmental boundaries and students would have the ability to “find” mentors based on the quality and vibrancy of their scientific program, the quality of their mentorship, and the stimulating supportive “ecosystem” of people and technology that their laboratories, and corresponding departments, provided. In this way, a form of natural selection occurred – those faculty, irrespective of seniority or physical location, who provided these attributes were chosen by students. The “fitness” of a lab was defined by these characteristics to the benefit of student, lab, department, school, and university.

The DBBS plan also foresaw the ultimate demise of the traditional departmental approach, opening more opportunities for training and collaboration. This was aptly illustrated by the demise of the NIH Training Grant Program in the second Nixon Administration that occurred shortly after the DBBS was formed. When these training grants were recast a year later for non‐departmental, integrated programs, their new requirements virtually detailed what Washington University had already put in place. Ultimately, it came to be widely known in graduate circles as the Wash U model.

Today, essentially all programs in biomedical science graduate education use a variation of this plan. One of the brilliant features of the model was an endowment (from a bequeathment to the university from the will of Edward Mallinckrodt) made available to an executive committee comprised of the basic science department heads of the medical school and the head of the Department of Biology. Committee members could leave the table and create their own departmental program, but the money remained, to be used only for the DBBS; this feature encouraged cooperation and, in the end, the success of the program. John Russell, long time director of the DBBS remembered a quote from Bill Danforth, “a vision without resources is hallucination.” Danforth provided the opportunity and garnered the resources that made the Division concept become a reality and it may well have had more impact on a greater number of people than any of his other accomplishments.

William Henry Danforth II was born on April 10, 1926, in St. Louis. Both his father, Donald, and his grandfather, William H., for whom he was named, were involved in the animal feed business that ultimately became Ralston Purina, one of the most important economic drivers of that city and the source of support behind the Danforth Foundation. Danforth, who began his undergraduate training at Westminster College in Fulton, MO during the Second World War, eventually graduated from Princeton with a degree in biology in 1947 and then attended Harvard Medical School. While studying in Boston, he met and married fellow St. Louisan, Elizabeth Gray, a student at Wellesley, in 1950, with whom he raised four children. She passed away in 2005. Following graduation from medical school in 1951, he interned at Barnes Hospital, served in the Navy during the Korean War, and then returned to St. Louis for good to a faculty position at Washington University School of Medicine in 1957. From there he rose through the administrative ranks of the medical school and then to the chancellorship.

He retired from this post in 1995, becoming Chancellor Emeritus, but continued as Chair of the Board of Trustees for many years thereafter. During this period of his life, he became heavily involved in plant science activities, the crowning achievement being the creation of the Donald Danforth Plant Sciences Center; he also was involved in other notable programs, many of which flourished as the result of the generosity of the Danforth Foundation, on whose Board he served from the mid 50s until it closed in 2011. He also continued his commitment to important community issues. In 1995, he was appointed to negotiate a settlement to the St. Louis Public School desegregation case and his leadership led to a solution that resulted in the bussing of thousands of African Americans living in St. Louis City and its underfunded schools, to St. Louis County where more educational resources were available. Bill Danforth died on September 16, 2020, at the age of 94 at his home in St. Louis.

There are many stories and anecdotes about Bill Danforth, particularly from his days as chancellor that illustrate his special prowess as an outstanding educator. He began this job as chancellor during trying times for American academia when college campuses were being severely disrupted by student dissatisfaction with the Viet Nam war and with violations of civil rights, particularly as manifested in the apartheid structure of South Africa. Indeed, it had been this type of unrest on the Washington University campus that had led his predecessor to retire, probably earlier than he would have. Danforth was not greeted with open arms by all at the outset. His familial connections to the business community were a concern for many (and this lasted for many years) and he lacked substantial academic administrative experience. But he had a “regular guy” persona that quickly dispelled the concerns of students and he rapidly became a highly respected and appreciated figure. Known widely among the student body as “Chan Dan” or “Uncle Bill,” affectionate sobriquets, he regularly made himself available for even casual functions on the campus. Illustrative of this approach, shortly before his retirement, Danforth appointed James McLeod as Dean of the College, the undergraduate liberal arts division, at Washington University. McLeod introduced a highly successful advising system in the college whose mantra was, “Every student to be known by name and story,” an advising system that, similar to the DBBS, was widely admired and copied by other colleges and universities.

**FIGURE 2:**
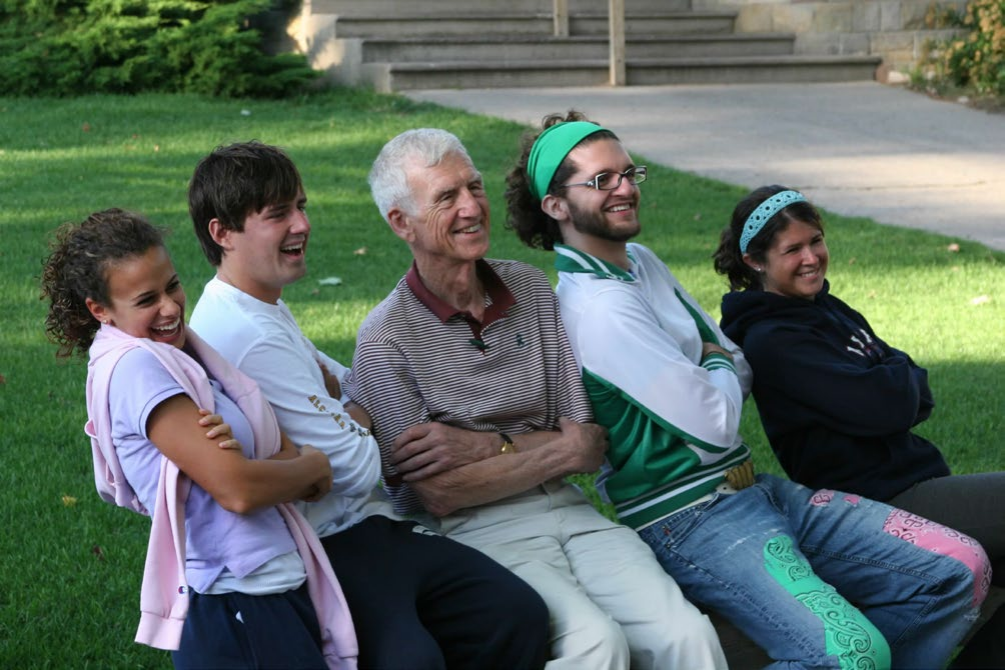
Washington University Danforth Scholars (l to r: Elizabeth Burack, Andy Flick, Christopher Kelly, and Laine Turkish) sharing a moment with Chancellor Emeritus Danforth (center) at Camp Miniwanca during student pre‐orientation. Photo courtesy of Philip D. Stahl. Used with permission

In recognition of Danforth’s contributions both to the University and the St. Louis community, upon his retirement, a scholarship fund was established at Washington University to recognize a cohort of outstanding entering students who were committed to service and community leadership along with humility, a signature characteristic of Danforth. This program was named the William H and Elizabeth Gray Danforth Scholars Program. Students selected for the Danforth Scholars Program begin their college experience with a trip to Camp Miniwanca, a camp founded in the 1920s by Danforth’s grandfather along with three other prominent American leaders. The camp adopted Native American tradition devoted to developing healthy attitudes about a balanced life and an appreciation of the natural environment. The former director of the program commented that the most impactful part of those days was Bill’s presence. He joined the students at camp where he shared his gentle wisdom and his perspective on making the world a little bit kinder (Figure 2). He was always giving, but from his perspective, it was others who provided him the opportunity, in the words of Camp Miniwanca’s motto, “to be his own self, at his very best, all the time.”

Bill Danforth made great contributions to the human experience to the benefit of all who knew and worked with him. Through his philanthropic role in the Danforth Foundation, he broadly helped and improved many organizations, and, because of his training, he was a particular champion of science. Most of all, as an educator and university leader, he impacted generations of students in helping them in their pursuit of knowledge. We, who had the privilege of being part of one of his many communities, the School of Medicine, the DBBS, the University faculty and student body, and educators everywhere, are richer for the experience.

